# Effects of Meteorological Factors on Hospitalizations in Adult Patients with Asthma: A Systematic Review

**DOI:** 10.1155/2019/3435103

**Published:** 2019-06-02

**Authors:** Elnaz Bodaghkhani, Masoud Mahdavian, Cameron MacLellan, Alison Farrell, Shabnam Asghari

**Affiliations:** ^1^Ph.D. Candidate, Centre for Rural Health Studies, Clinical Epidemiology Department, Faculty of Medicine, Memorial University of Newfoundland, St. John's, Canada; ^2^MD, MSc, FACP, ABPM, FRCPC, Respirologist, Royal Victoria Regional Health Centre, Barrie, Ontario, Canada; ^3^M.Sc. Candidate, Centre for Rural Health Studies, Clinical Epidemiology Department, Faculty of Medicine, Memorial University of Newfoundland, St. John's, Canada; ^4^MLIS, Public Services Librarian Health Sciences Library, Memorial University of Newfoundland, St. John's, Canada; ^5^MD, MPH, Ph.D., Associate Professor, Director of Centre for Rural Health Studies, Faculty of Medicine, Memorial University of Newfoundland, St. John's, Canada

## Abstract

**Background:**

Environmental factors such as weather variables contribute to asthma exacerbation. The impact of meteorological factors on asthma-related hospital admissions (HAs) or emergency department visits (EDVs) has been assessed in the literature. We conducted a systematic review to establish a conclusion of whether these findings from the literature are consistent and generalizable or if they vary significantly by certain subgroups.

**Objective:**

This study aims to review the effect of meteorological variables on asthma HAs and EDVs in adults, to identify knowledge gaps and to highlight future research priorities.

**Method:**

A systematic search was conducted in electronic databases such as PubMed, Embase, and CINAHL. All studies published in English were screened and included if they met the eligibility criteria. Two independent reviewers assessed the quality of the studies and extracted the data. The available evidence was summarized and presented using a harvest plot.

**Results:**

Our initial search returned a total of 3887 articles. After screening titles, abstracts, and full texts, 16 studies were included. Thirty-one percent of the included studies (5/16) found that temperature was the only factor associated with asthma hospitalization or EDVs. Six studies (37%) found that both temperature and relative humidity were associated with HAs. Four studies (25%) identified thunderstorms as a possible factor associated with asthma hospitalization in adults.

**Conclusion:**

Our review suggests that HAs and EDVs due to asthma are associated with many meteorological factors. Among the articles included in this review, changing temperature is the most commonly studied variable. We did not find studies that measured barometric pressure, weather phenomena, or the effect of tornados. To develop effective strategies to protect subjects at risk, further studies are required.

## 1. Introduction

Asthma is a chronic inflammatory condition which is characterized by reversible air flow obstruction [1]. Those affected by this condition may present symptoms such as wheezing, breathlessness, coughing, and chest tightness. This chronic condition is highly prevalent, and according to the World Health Organization (WHO), 300 million people around the world have asthma; it is estimated that 100 million more people will be affected by 2025 [2]. While the etiology of asthma is currently unknown, it is believed that factors such as genetics, lifestyle choices, and environmental factors contribute to asthma exacerbation which may lead to HAs [3].

Among environmental factors, weather and air pollution play an important role in increasing asthma exacerbations, which require special care or even hospitalization [4]. Many studies including systematic reviews assessed the effect of pollen, allergens, and air pollutions on asthma hospitalization. While many studies indicate that weather factors are positively associated with HAs due to asthma, the exact mechanism is unclear [1, 5, 6]. Some studies found temperature and humidity could have an influence on airway function and might affect lung function [7]. One theory suggests that water loss in airways during cold conditions causes inflammation that leads to asthma attacks [2]. Another theory suggests that cold and dry air increases the rate of evaporation of surface fluid in airways [8]. Furthermore, some studies show that changes among weather variables can lead to airway inflammation among people with asthma [3].

Some studies suggest that extreme temperature (both cold and hot) and high winds could increase EDVs and hospitalizations among those with asthma [9, 10]. In contrast, other authors did not find any correlation between weather variables and asthma HAs [11]. To our knowledge, there is no review that summarizes the evidence on the effects of meteorological variables on HAs and EDVs among adults with asthma. This review could be important for healthcare demand prediction during weather events. The objectives of this review are (1) to summarize and evaluate the effects of meteorological variables on asthma hospitalization and EDVs in adults, (2) to identify knowledge gaps, and (3) to highlight future research priorities.

## 2. Methods

### 2.1. Review Question

Our systematic review question “Do weather conditions affect HAs among adults living with asthma?” was defined using the PECO question format where the study *P*opulation is adults (age ≥18 years) who were diagnosed with asthma according to the asthma definition in the study. *E*xposure of interest includes any weather variables reported in the study. The *C*ontrol groups are adults with asthma who were not exposed to weather variables, and the *O*utcome is any admission to hospital units or emergency departments as reported in the manuscripts.

### 2.2. Data Sources

We searched large electronic databases containing health and medical literature: PubMed (1946 to December 2018), Embase via Embase.com (1947 to December 2018) and CINAHL via Ebsco (1937 to December 2018). Moreover, references of included articles were searched, and the articles deemed eligible were included in the review. The search was originally done in November 2017 and updated to include articles published until December 31, 2018.

### 2.3. Search Strategy

A series of relevant terms were identified through consultation between content experts and a librarian. The keywords and Medical Subject Headings (MeSH) for this review fall under the following categories: (1) asthma, (2) hospitalization, and (3) meteorological factors. We included a combination of keywords such as temperature, precipitation, thunderstorm, wind speed, and air pressure that could potentially influence asthma hospitalizations and EDVs (see Table 1, a sample search strategy and the key words we used in PubMed).

### 2.4. Inclusion Criteria

Studies included in this review consisted of original research papers from peer-reviewed journals and those published in the English language. We identified studies that focused on HAs or EDVs due to weather factors and looked for adult patients diagnosed with asthma.

Dissertations, audits, policy analyses, book reviews, pilot studies, perspective articles, and research at the planning stage (unless provided in the research directory) were excluded. We also excluded studies that evaluated air pollution as the only exposure and those that used a pediatric population as children respond differently than adults to weather changes.

### 2.5. Directory of Identified Studies

A directory of publications was created in RefWorks. We used Mendeley to capture and tag the web pages' information, which we then imported into RefWorks.

### 2.6. Study Selection

The eligibility of articles included in this review was assessed in two stages:

#### 2.6.1. Stage 1: Prescreening

After removing duplicates, the titles and abstracts were screened by one reviewer (EB). We then selected articles that were deemed eligible for full-text review. A random selection of five percent of the articles was assessed by a second reviewer (CM) to assess the validity of the screening. There was 98% agreement, so a second review of the titles and abstracts was unnecessary.

#### 2.6.2. Stage 2: Full-Text Review

**Figure 1:**
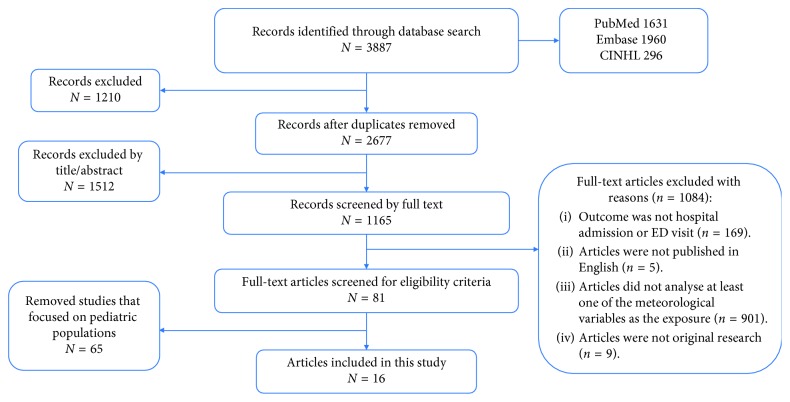
Preferred Reporting items for Systematic Reviews (PRISMA) diagram.

We performed a full text review of relevant articles found in the prescreening phase. Two reviewers (EB and CM) independently checked the full-texts of the articles against the inclusion criteria and fine-tuned the final article selection for data extraction. Any discrepancies were discussed and resolved.  Figure 1 shows our PRISMA diagram.

### 2.7. Data Extraction

A data extraction tool was developed using Excel. The data extraction tool included the following six characteristics: citations (e.g., authors, publication year, journal, and place of study), methodology (e.g., study design and sample size), characteristics of study population (e.g., location of study), meteorological factors (e.g., precipitation, thunderstorms, humidity, and temperature), hospitalization (e.g., HAs and EDVs), and study quality.

Data extraction was conducted by two independent reviewers (EB and CM). A calibration test was performed on the first 10% of the reviewed studies. The Kappa index was calculated to assess inter-reviewer agreement (kappa >0.7 was considered a good agreement). The discordant items were reassessed, and the data extraction tool was revised accordingly. The reviewers met weekly to discuss the extracted data and resolve any disagreements. A third reviewer (SA) was invited to mediate if the disagreement did not resolve.

### 2.8. Quality Assessment

We used the Critical Appraisal Skills Program (CASP, 2014) tool to assess the quality and risk of bias of included studies. The tool assesses the study design, population of study, sample size, statistical methods, confounding variables, and biases. We did not exclude low-quality studies from this review, but we kept into account this aspect when summarizing the results.

### 2.9. Synthesis of Results and Data Display

We described all studies that met the eligibility criteria including their quality score. We quantified the frequency of the reported meteorological variables and summarized their effects on HAs or EDVs separately.

**Figure 2:**
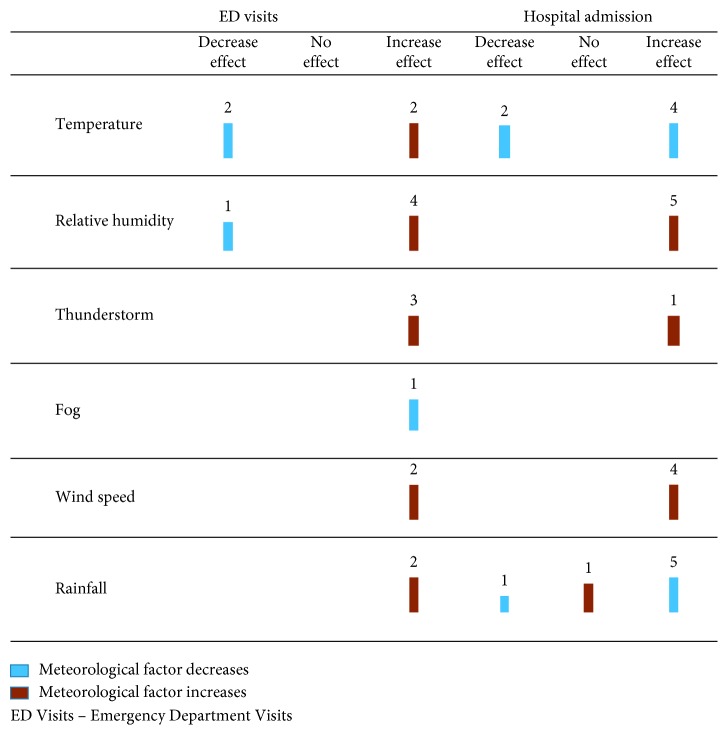
Evidence for the effect of meteorological factors on hospitalization and EDVs among adults with asthma. The rows indicate the all meteorological variables that affect asthma-related admission which are studied in the literature, and three columns shows different effects of each exposure. The numbers on the top of each bar indicate the number of studies that investigate the effect of that variable and find the result. The length of each bar shows the quality (good, fair, and poor) of the studies. The colors of each indicate the increase or decrease effect of the variable.

We developed a harvest plot to present our systematic review results (Figure 2). A harvest plot is a novel approach to graphically summarize the results of a systematic review of complex and diverse studies [12]. The harvest plot method is flexible by allowing us to display the quantitative data for all studies when it would not be possible to combine in a traditional forest plot [13]. The results of our systematic review show three outcomes (increasing effect, decreasing effect, and no effect) and six weather variables as the exposure which includes temperature, wind speed, fog, rainfall, thunderstorms, and relative humidity. Each row of the plot shows the different exposures examined in these studies. All plots contain a digit at the top of the vertical bar representing the number of studies that examined the factors for the outcome of interest. Each vertical bar of the plot was colored to indicate the effect of that variable on asthma-related admissions (increasing effect, decreasing effect, and no effect) and the size of bar indicates the quality of studies (good, fair, and poor).

## 3. Results

Our search for asthma-related HAs due to weather factors returned a total of 2677 articles after deduplication. 1512 studies were excluded while screening for titles and abstracts. During full-text review, five studies were excluded because they were not published in English and nine more studies were excluded because they were not original research articles. Eighty-one articles included either adult or pediatric populations. Among them, 65 were excluded as the study population was only children, resulting in 16 studies to be included in this review.  Figure 1 shows the PRISMA flow diagram of the included studies.

### 3.1. Study Characteristics

Seven of the 16 included studies were conducted in Asia (five in East Asia), five in the USA, two in Europe, and two in Australia (Table 2). Overall, these 16 studies covered 1,144,859 observations, where most articles included at least 50,000 observations each. The mean study period for included studies was 6.5 years with a standard deviation of 7.13 years. Aside from the single study with a case-control design, one ecological study, and one cohort study, eight studies in this review used time-series analysis and three conducted case-crossover designs. Articles included in this study used data sources from a health department database (*n*=9), hospital admission records (*n*=5), records from an insurance company (*n*=1), and a dataset from a fire department in Japan. All studies compared the individuals before and after they were exposed to weather conditions. There were no external comparison groups.

### 3.2. Weather Variables

As shown in Tables 3 and 4, 31% (5/16) of the studies found that temperature was the only factor associated with asthma hospitalization or EDVs [4, 9, 11, 15, 18, 25]. Nearly 37% (6/16) of the studies found that both temperature and relative humidity were associated with HAs (Table 4) [6, 9, 16, 18, 21, 22]. Twenty-five percent (4/16) investigated the effect of thunderstorms as a possible element for asthma hospitalization in adults [14, 17, 23, 24]. We did not find any studies that measured weather variables such as barometric pressure, different types of storms (tropical storm, and snow storm), and tornados.

### 3.3. Effect of Meteorological Factors on Hospitalization

Only one study showed a negative correlation between asthma hospitalization and daily mean temperature (5.79% risk increase, *p*=0.012) and lower minimum temperature (2.88% risk increase, *p*=0.024) during the cold season [19]. This study was conducted in Hong Kong and was adjusted for air pollution, solar radiation, and day of the week. Two studies, one in Finland (*r* = −0.11, *p* < 0.01) [22] and one in China (*r* = −0.174, *p* < 0.001) [9], have reported that asthmatic symptoms that lead to admission are influenced by daily temperature change during the study period. Five studies found that extremely cold or hot temperatures could trigger asthma attacks that lead to HAs [11, 14, 15, 16, 22, 29].

### 3.4. Effect of Meteorological Factors on EDVs

One study reported that during the summer months, EDVs for asthmatic patients had increased with increasing temperature and humidity [22]. Lower relative humidity was the cause of the increase in EDVs among asthmatic patients in another study [18]. One study [17] reported that rainfall events due to thunderstorms could increase the EDVs for asthmatic patients (Figure 2).

## 4. Discussion

Our review suggests that temperature variation can have both a positive or negative correlation with asthma hospital admissions, depending on the season and geographic area. The temperature measurement and the threshold for temperature vary from one study to another. Moreover, temperature itself could be influenced by other variables (e.g., wind speed and barometric pressure) which were not taken into account by many studies. Aside from temperature, other weather variables including relative humidity and precipitation [18, 19, 20], wind speed [17], and thunderstorms [24] could influence asthmatic patients' condition and the number of HAs.

This study suggests that changes in weather variables could increase EDVs due to asthma. Temperature variation was the most frequently studied factor among the included studies. Thunderstorms [17], rainfall events [17, 27], low temperatures [6, 21], increase in humidity [19], and fungal spores [28] were other variables that could increase EDVs among patients living with this chronic condition. Kwon et al. described a negative relationship between the incidence of fog and EDVs [18]. It is important to note that the definition of weather variables and EDVs have not been consistent across all studies.

Our review has some limitations. First, many studies included in this review did not adjust for the effect of air pollution and pollens where the effect of weather variables was assessed. Air pollution and pollens are known risk factors for asthma exacerbation [27]. A review by D'Amato et al. recommends that changes in weather factors could influence the rate of asthma attacks depending on the intensity and length of the pollen season. Another study by the same author reported asthma exacerbations due to the increased pollen in the air during thunderstorms [29].

Other studies indicated a significant increase in the number of patients with bronchial asthma visiting an emergency clinic during December due to fungal spores [20]. A systematic review by Zheng et al. identified associations between several air pollutants and EDVs due to asthma. This study also showed that weather factors such as wind speed and direction play a key role in air pollution [30].

Only peer-reviewed articles published in English were included, making it possible that we missed studies published in other languages or in the grey literature.

Although there were no limitations to our study design or data collection methods, the studies included in this review are mainly observational studies (time-series and case-crossover studies) using secondary data where the data quality is a concern [28, 31, 32].

To our knowledge, this is the first systematic review assessing the effects of meteorological factors on asthma hospitalizations. We identified only 16 studies, 6.25% (1/16) with poor quality, suggesting a lack of available evidence. Estimating a summary effect of meteorological factors on asthma hospitalizations among adults was not possible due to heterogeneity across the studies.

## 5. Conclusion

Information on how particular weather events, such as extreme wind or cold, could affect asthma HAs is essential to predict hospital demands and to help prevent exacerbation among patients with asthma and its consequent HA. Our review shows that asthma HAs and EDVs in adults are associated with temperature variability. It also suggests that weather variables including relative humidity, rainfall, and wind are factors which influence EDVs and hospitalization among patients living with asthma. Our study suggests the possibility of a gap in the knowledge regarding the effect of barometric pressure, weather phenomena, and tornados. Due to inconsistencies of the methodological approaches and the differences in statistical analyses in the included articles, we were not able to generate a practical policy recommendation. Methodological limitations of the studies and inconsistencies in the study findings show great potential for future research.

## Figures and Tables

**Table 1:** Literature search strategy for PubMed and number of identified articles.

1	“asthma” [MeSH Terms] OR “asthma” [All Fields] OR (“chronic” [All Fields] AND “respiratory” [All Fields] AND “disease” [All Fields]) OR “chronic respiratory disease” [All Fields] OR “asthma” [Mesh]	223989

2	“hospitalization” [Mesh] OR “emergency service, hospital” [Mesh] OR ((“hospitals” [MeSH terms] OR “hospitals” [All fields] OR “hospital” [All fields]) AND admission [All fields]) OR (“length of stay” [MeSH terms] OR (“length” [All fields] AND “stay” [All fields]) OR “length of stay” [All fields] OR (“hospital” [All fields] AND “stay” [All fields]) OR “hospital stay” [All fields]) OR ((“hospitals” [MeSH terms] OR “hospitals” [All fields] OR “hospital” [All fields]) AND admissions [All fields]) OR (“length of stay” [MeSH terms] OR (“length” [All fields] AND “stay” [All fields]) OR “length of stay” [All fields] OR (“hospital” [All fields] AND “stays” [All fields]) OR “hospital stays” [All fields]) OR exacerbation [All fields] OR exacerbations [All fields]	481723

3	“weather” [Mesh] OR meteorological [All fields] OR (“weather” [MeSH terms] OR “weather” [All fields]) OR (“temperature” [MeSH terms] OR “temperature” [All fields]) OR (“humidity” [MeSH terms] OR “humidity” [All fields]) OR (“wind” [MeSH terms] OR “wind” [All fields]) OR (“rain” [MeSH terms] OR “rain” [All fields]) OR (“snow” [MeSH terms] OR “snow” [All fields]) OR precipitation [All fields] OR thunder [All fields] OR (“lightning” [MeSH terms] OR “lightning” [All fields]) OR storm [All fields] OR (“cyclonic storms” [MeSH terms] OR (“cyclonic” [All fields] AND “storms” [All fields]) OR “cyclonic storms” [All fields] OR “Hurricane” [All fields]) OR (“tornadoes” [MeSH terms] OR “tornadoes” [All fields] OR “tornado” [All fields]) OR (“droughts” [MeSH terms] OR “droughts” [All fields] OR “drought” [All fields]) OR “meteorological concepts” [Mesh] OR (“atmosphere” [MeSH terms] OR “atmosphere” [All fields]) OR atmospheric [All fields] OR “air pressure” [All fields] OR (“climate” [MeSH terms] OR “climate” [All fields]) OR (“seasons” [MeSH terms] OR “seasons” [All fields]) OR seasonal [All fields] OR “thunderstorm” [All fields] OR thunderstorms [All fields]	1298001

4	1 AND 2 AND 3	1631

5	4 AND publication date to Nov. 17, 2017	1511

6	4 AND publication date Nov. 17, 2017–Dec. 31, 2018	120

**Table 2:** Characteristics of studies that examine the effect of meteorological factors on asthma admissions.

Author	Objective	Study design	Data source		Measures of effect			Study outcome		Quality
			Health data	Climate data	OR	RR	Count	EDV^1^	HA^2^	
Abe et al. [6]	Investigate the relationship of weather conditions and asthma exacerbation	Time-series	Tokyo Fire Department, follow-up diagnostic data from emergency physicians	Japan Meteorological Agency	X			X		Good
Anderson et al. [14]	Investigate associations between asthma admissions and thunderstorms	Case control	Computerized hospital record	Met. Office and Cardiff Airport measurement site.			X		X	Fair
Buckley and Richardson [15]	Characterize the effect of temperature on EDVs for asthma	Case-crossover	Epidemiologic Collection Tool (NC DETECT)	State Climate Office of North Carolina	X			X		Fair
Delamater et al. [16]³	Investigate the relationships between air pollution, weather conditions, and asthma hospitalizations	Ecological	Healthcare Information Resource Center	Environmental Protection Agency (EPA)			X		X	Fair
Fitzgerald et al. [11]	Investigate whether prolonged periods of very cold temperatures are associated with an increased risk of hospitalization for asthma patients	Time-series	New York State Department of Health, Statewide Planning and Research Cooperative System (SPARCS)	National Center for Atmospheric Research			X		X	Fair
Grundstein et al. [17]	Examine the association between thunderstorm activity and asthma morbidity	Time-series	EDV database	Automated surface observing system station		X		X		Poor
Kunikullaya et al., 2017³	Determine the relationship between acute exacerbations of asthma and related HAs due to air pollution and meteorological conditions	Retrospective ecological time-series	Admission recorded by the hospital	Central laboratory of Karnataka State Pollution Control Board and meteorological department			X	X		Fair
Kwon et al. [18]³	Estimate the effect of climate factors and air pollution on asthma hospitalization	Case-crossover	Kangwon National University Hospital and Chuncheon Sacred Heart Hospital	Database of the Korea Meteorological Administration		X		X		Fair
Lam et al. [19]	Evaluate associations between asthma hospitalizations and meteorological factors in Hong Kong.	Time-series	Hospital authority	Single central monitoring station from the Hong Kong Observatory (HKO)			X		X	Fair
Qasem et al. [20]³	Explore which weather factors contribute to asthma hospitalization while controlling for pollen and spore level in the air in Kuwait	Retrospective time-series study	Medical records from two hospitals (Al-Rashid Allergy Center and Emergency Department, and Al-Sabah Hospital)	Kuwait Aviation/Meteorology Department			X		X	Fair
Qiu et al. [21]	Examine the health effects of environmental triggers on asthma	Longitudinal time-series	Hospital Authority Corporate Data Warehouse	Hong Kong Observatory			X	X		Fair
Rossi et al. [22]³	Evaluate the relationships between EDVs for asthma attacks and the meteorological, aerobiological, and chemical characteristics of the outdoor air	Time-series	University Central Hospital	Measured at the meteorological station in the city of Oulu			X	X		Fair
Soneja et al., 2016	Investigate the association between exposure to extreme heat and precipitation events and risk of hospitalization for asthma	Case-crossover	Maryland Department of Health and Mental Hygiene	National Climatic Data Center		X			X	Fair
Zhang et al. [9]	Evaluate the short-term effects of daily mean temperature on asthma HAs.	Time-series	Health Insurance System of Shanghai	Shanghai Center for Urban Environmental Meteorology		X	X		X	Good
Andrew et al. [23]	Assess the demand for emergency medical services during epidemic thunderstorm asthma	Time-series	Ambulance Victoria data warehouse and emergency service telecommunication	Australian Bureau of Meteorology				X		Good
Thien et al. [24]	Investigate the effect of thunderstorm asthma on health services and patient risk factors	Cross-sectional	Ambulance Victoria, the Victorian Department of Health and Human Services Victorian, Australian and New Zealand Intensive Care Society Adult Patient Database, census data	Australian Bureau of Meteorology						

^1^EDVs: emergency department visits for asthma. ^2^HAs: hospital admissions for asthma. ^3^Air pollution was considered a confounder in these studies.

**Table 3:** Studies that examine the effect of meteorological factors on EDVs.

Meteorological risk factors											
Location		Author	Sample size	Temperature	Relative humidity	Thunderstorm	Fog	Wind speed	Rainfall	Key measures	Results
*North America*											
North Carolina, USA		Buckley and Richardson. [15]	53, 156	YES						Daily min./max. temperature	OR for EDVs per 278.15° *K* = 1.01, 95% CI: 1.00–1.02
Atlanta, USA		Grundstein et al. [17]	215, 832			YES		YES	YES	Total daily rainfall	EDVs 3% higher on days following thunderstorm

*Europe*											
Oulu, Finland	Rossi et al. [22]		232	YES	YES				YES	Min./max. and mean temperature, relative humidity, rainfall	Increased EDVs during the summer due to higher temperature and humidity, (*r* = −0.11, *p* < 0.01)

*East Asia*											
Chuncheon, Korea	Kwon et al. [18]		660	YES	YES		YES	YES	YES	Max./min./mean temp., temperature range, low and mean relative humidity, rainfall, fog present	Low relative humidity increased and fog decreased EDVs. Risk increase: 29.4% (95% CI: −46.3% to −7.2%, *p*=0.013)
Tokyo, Japan	Abe et al. [6]		643, 849	YES	YES				YES	Min. temperature and max. relative humidity. Total rainfall	Lower temperature increases EDV by % 1.2
Hong Kong	Qiu et al. [21]		45, 896	YES	YES					Daily diurnal temperature range	274.15°K in diurnal temperature range associated with a 2.49% (95% CI: 1.86% to 3.14%) increase in daily EDVs
Victoria, Australia	Andrew et al. [23]		2954	YES		YES				Dropping temperature	41.7% (95% CI: 39.6% to 43.9%) increase in ER visits due to thunderstorm
Melbourne, Australia	Thien et al. [24]		3365	YES	YES	YES				Plunging temperature and rising humidity	992% increase in asthma-related EDVs

**Table 4:** Studies that examine the effect of meteorological factors on HAs.

Meteorological risk factors									
Location	Author	Sample size	Temperature	Relative humidity	Thunderstorm	Wind speed	Rainfall	Key measures	Results
*Europe*									
Cardiff and Newport, UK	Anderson et al. [14]	2000			YES		YES	Min./max. temperature and total daily rainfall	Average daily asthma hospitalization was lower during the summer (4.1, May–September, *p*=0.04). More admissions occurred during thunderstorms (*p*=0.04); however, there was no relationship between rainfall and admissions

*North America*									
Los Angeles, USA	Delamater et al. [16]	250, 000	YES	YES				Max. temperature and relative humidity	HAs increased during winter (0.481 per 100,000 admissions)
New York, USA	Fitzgerald et al. [11]	237, 639	YES			YES		Cold spells lasting three days, where the daily mean temperature was less than the 10th percentile for a given month and region	HAs increased in November (mean = 9.6, 95% CI: 5.5% to 13.9%) and April (mean = 5.0, 95% CI: 1.2% to 9.0%)
Maryland, USA	Soneja et al., 2016	115, 923	YES				YES	Daily max. temperature and total daily precipitation	Extreme heat increased HAs by 3% (OR: 1.03, 95% CI: 1.00 to 1.07)

*South Asia*									
Bangalore, India	Kunikullaya et al., 2017	1768	YES	YES			YES	Max./min./average temp., relative humidity, and total daily rainfall	Average daily asthma admission was 4.84 ± 2.91, had seasonal variation and increased during the cold season (*p*=0.015)

*Middle East*									
Kuwait	Qasem et al. [20]	4353	YES	YES		YES	YES	Daily temperature, relative humidity, and total daily rainfall	Hospitalization increased during December due to high temperatures (mean = 39.7, *p* < 0.03)

*East Asia*									
Shanghai, China	Zhang et al. [9]	15, 678	YES	YES		YES	YES	Min./max. and mean temperature, relative humidity, and total daily rainfall	RR: 1.20 (95% CI: 1.01 to 1.41) for lower temperatures
Hong Kong	Lam et al. [19]	56, 112	YES	YES		YES		Daily mean temperature and mean relative humidity	Cumulative risk of hospitalizations during the hot season was 1.19 (95% CI: 1.06 to 1.34)
